# Administration of the vasopressin analog desmopressin for the management of bleeding in rectal cancer patients: results of a phase I/II trial

**DOI:** 10.1007/s10637-020-00914-5

**Published:** 2020-03-12

**Authors:** Soledad Iseas, Enrique L. Roca, Juan M. O’Connor, Martin Eleta, Analia Sanchez-Luceros, Daniela Di Leo, Marcelo Tinelli, Maria L. Fara, Eduardo Spitzer, Ignacio A. Demarco, Giselle V. Ripoll, Marina Pifano, Juan Garona, Daniel F. Alonso

**Affiliations:** 1 Oncology Unit, Gastroenterology Hospital Bonorino Udaondo, Buenos Aires, Argentina; 2 Department of Clinical Oncology, Alexander Fleming Institute, Buenos Aires, Argentina; 3 Onco-imaging Area, Imaxe Center for Diagnostic, Buenos Aires, Argentina; 4 Thrombosis and Hemostasis Department, IMEX and IIHEMA, National Academy of Medicine, Buenos Aires, Argentina; 5 Elea-Phoenix Laboratories, Los Polvorines, Argentina; 6 Chemo-Romikin, Buenos Aires, Argentina; 7 Laboratory of Molecular Oncology, National University of Quilmes, Bernal, Argentina

**Keywords:** Vasopressin peptide analog, Drug repurposing, Tumor perfusion, Hemostasis, von Willebrand factor, Gastrointestinal cancer

## Abstract

*Purpose* The vasopressin analog desmopressin (dDAVP) is known to increase plasma levels of hemostatic factors, and preclinical studies in colorectal cancer models have demonstrated that it hampers tumor vascularization and metastatic progression. We evaluated safety and preliminary efficacy of dDAVP in rectal cancer patients with bleeding, before receiving specific oncologic treatment with surgery, chemotherapy and/or radiotherapy. *Methods* Patients with rectal cancer having moderate or severe rectal bleeding were enrolled in an open-label, dose-finding trial. Intravenous infusions of dDAVP were administered during two consecutive days in doses from 0.25 to 2.0 µg/kg, using single or twice daily regimen. Bleeding was graded using a score based on the Chutkan scale and tumor perfusion was evaluated by dynamic contrast-enhanced magnetic resonance imaging. *Results* The trial accrued a total of 32 patients. Dose-limiting toxicity occurred in patients receiving 1 µg/kg or higher. The most prominent treatment-related severe adverse event was hyponatremia. Most patients receiving the maximum tolerated dose of 0.5 µg/kg showed at least a partial hemostatic response and 58% developed a complete response with absence of bleeding at day 4 and/or at the last follow-up at day 14. Tumor perfusion was decreased in two-thirds of patients after dDAVP treatment. *Conclusions* dDAVP appeared as a promising hemostatic agent in rectal cancer patients with bleeding. Randomized clinical trials to confirm its effectiveness are warranted.

**Clinical trial registration** www.clinicaltrials.gov NCT01623206

## Introduction

Colorectal cancer is one of the most common cancers worldwide, resulting in more than half a million deaths every year. While early-stage disease has a survival rate over 90%, advanced colorectal cancer cannot be cured in most cases, requiring multidisciplinary care from surgeons, oncologists and palliative care practitioners [1]. In the case of rectal cancer, multimodal management has changed the approach of locally advanced lesions, complementing surgery with radiotherapy and chemotherapy [2]. Neoadjuvant therapy has shown promising results with remarkable responses to preoperative chemoradiation, and combined-modality therapy is recommended for most patients with stage II or III rectal cancer [3, 4]. However, symptoms of mucorrhea, tenesmus, rectal pain and especially rectal bleeding are difficult to treat and have a direct impact on quality of life. On some occasions, during diagnostic work-up or in a palliative setting, symptomatic relief of bleeding is required. Different treatments aimed at the reduction of rectal blood loss and accompanying symptoms have been evaluated, although most available data come from studies about the management of radiation proctitis [5, 6].

Desmopressin (1-deamino-8-D-arginine vasopressin, also known as dDAVP) is a synthetic peptide derivative of the hormone vasopressin, being a selective agonist for the vasopressin V2 membrane receptor (AVPR2). The antidiuretic effect of the compound is mediated by renal AVPR2, whereas activation of AVPR2 present on microvascular endothelial cells by dDAVP causes the release of von Willebrand factor (VWF) and coagulation factor VIII (FVIII), as well as the profibrinolytic enzyme tissue-type plasminogen activator [7]. The potent hemostatic effects of dDAVP at relatively low doses of 0.2–0.3 µg/kg allows its use in the management of bleeding disorders and as a blood-saving agent during surgical procedures [8]. In addition, preclinical evidence has been accumulating on the antiangiogenic and antimetastatic properties of dDAVP at a higher dose range of 1–2 µg/kg in animal models of breast, prostate and colorectal cancer [9–13]. A recent clinical trial in breast cancer patients administered with perioperative infusions of dDAVP demonstrated a reduced intrasurgical bleeding with increasing doses of the compound. Interestingly, treatment was associated with higher VWF plasma levels and a postoperative decrease in circulating tumor cell counts [14].

Seminal studies by Mannucci and coworkers [15, 16] in the late seventies demonstrated good tolerance and hemostatic efficacy of intravenous dDAVP in healthy volunteers and patients with hemophilia A or von Willebrand’s disease. Clinical indications for dDAVP quickly expanded to include acquired and congenital platelet disorders and other hemostatic abnormalities. The broadening use of dDAVP had led investigators to explore its value in other settings, such us surgical operations associated with significant blood loss or transfusion requirement [17]. Recently, a large study in a cohort of colorectal cancer patients demonstrated that blood transfusion is associated with worse prognosis after curative tumor resection [18]. Similarly, the need for blood transfusion was shown to be a predictor of 2-year mortality in patients diagnosed with rectal cancer undergoing surgery [19], suggesting that blood-saving measures could have a favorable impact on prognosis.

Considering the well-known hemostatic properties and pharmacologic profile of dDAVP as well as its potential antitumor activity, we conducted a phase I/II in rectal cancer patients with bleeding, administering a lyophilized formulation of dDAVP by intravenous infusion in saline, before receiving specific oncologic treatment with surgery, chemotherapy and/or radiotherapy. The study consisted of a dose escalation design (phase I) followed by an expansion cohort to investigate early efficacy on bleeding (phase II).

## Methods

### Patient selection

Patients were enrolled from the Gastroenterology Hospital Bonorino Udaondo and the Alexander Fleming Institute, Buenos Aires (Argentina). Eligible patients were male and non-pregnant non-lactating female subjects, aged between 18 and 80 years, with histological diagnosis of local, locally advanced or metastatic rectal cancer, having moderate or severe rectal bleeding associated to primary tumor. After hemostatic therapy, patients had the indication of surgical resection and/or chemotherapy and/or radiotherapy. Exclusion criteria included known hypersensitivity to dDAVP or vasopressin, hormone therapy, severe von Willebrand´s disease or hemophilia, syndrome of inadequate secretion of antidiuretic hormone, renal impairment or hyponatremia, prior history of seizures, congestive heart failure, blood hypertension, heart arrhythmia, thromboembolic disease, diabetes type I or II, liver disease, positive serology for hepatitis B or C and/or human immunodeficiency virus infection, any active infection that would affect patient safety and other malignant diseases.

### Study design

This was an open-label, dose-finding, phase I/II trial. The primary endpoint of the phase I part of the study was to select the best dDAVP dose for use in rectal bleeding, while of the phase II part was to preliminary evaluate the efficacy in terms of hemostatic results in patients with rectal cancer. Secondary endpoints included safety, tolerability, evaluation of rectal bleeding by a score, assessment of tumor perfusion and biochemical analysis of blood.

All patients provided written informed consent before enrollment. The study protocol was approved by the ethics committee at each site and by the National Administration of Drugs, Food and Medical Technology of Argentina (ANMAT Disp. 1506). The study was conducted according to the Declaration of Helsinki and the principles of good clinical practice. The trial was registered at ClinicalTrials.gov (NCT01623206).

### Safety assessments

Safety and tolerability were assessed for all enrolled patients from the time the patient signed the informed consent through post-treatment follow-up. Adverse events were graded according to the NCI Common Toxicity Criteria for Adverse Events (CTCAE, Version 4.0) [20]. Severe adverse events (SAEs) were reported to the sponsor and the ethics committees, and were followed up until resolution.

### Administration of study treatment

Eligible patients were administered with intravenous infusions of dDAVP during two consecutive days. A lyophilized formulation of dDAVP (Surprex TM, Elea-Phoenix Laboratories, Buenos Aires, Argentina) was diluted in 100 mL of saline solution and slowly infused over the course of approximately 20–30 minutes. Dose escalation was conducted following a classical 3 + 3 dose-escalation design [21] in which five treatment groups of at least 3 patients each received increasing dDAVP daily doses from 0.25 to 2.0 µg/kg, according to the scheme in Table 1. Dose-limiting toxicity (DLT) was defined as any grade 3 or 4 treatment-related adverse event within 14 days after starting dDAVP administration. The maximum tolerated dose (MTD) was defined as the dose level below the lowest dose that induced DLT in at least one-third of patients (at least 2 of a maximum of 6 patients). If no DLT occurred, dosages were escalated to the next group of patients. If DLT occurred in at least 2 patients at a given dose level, then the next 3 patients were treated at the next lower dose level. If DLT occurred in 1 of 3 patients treated at a given dose level, 3 additional patients were enrolled and treated at the same dose level. If DLT occurred in at least 1 of these additional 3 patients the MTD was exceeded, and the MTD was defined as the previous dose unless only 3 patients were treated at that dose level. In that case, 3 additional patients were treated at that lower dose level. After MTD was defined, an expansion cohort of 12 patients was enrolled to receive the selected dose of dDAVP (see also Table 1). Paracetamol (acetaminophen) as concomitant medication was allowed for symptom management, as well as tramadol or methadone in patients with very severe pain. Patients did not receive tranexamic acid, aspirin or other non-steroidal anti-inflammatory drugs during or following dDAVP treatment until the last follow-up at day 14.

**Table 1:** Treatment groups, dosage and schedule of administration of dDAVP

Group	Individual dose^a^ (µg/kg)	Total daily dose (µg/kg)
Dose level 1 Dose level 2 Dose level 3 Dose level 4 Dose level 5	0.25 per day 0.25 per 12 hours 0.5 per 12 hours 1.0 per day 1.0 per 12 hours	0.25 0.5 1.0 1.0 2.0
Expansion cohort	0.5 per day	0.5

^a^dDAVP was administered through intravenous infusions for 2 consecutive days.

### Evaluation of rectal bleeding

Rectal bleeding was graded using a score from 0 to 10 points, where 0 is absent, 1–3 is mild, 4–7 is moderate and 8–10 is severe. A partial hemostatic response (PR) was defined as a reduction in at least one grade, and a complete response (CR) corresponded to total disappearance of symptoms. The score was obtained by adding the points for the severity of bleeding according to the Chutkan scale [5] (0 = no blood; 1 = blood on toilet paper or stool; 2 = blood in toilet bowl; 3 = heavy bleeding with clots; 4 = bleeding requiring transfusion), the number of daily episodes (0 = no episodes; 1 = 1–3 episodes; 2 = 4–6 episodes; 3 = 7–10 episodes; 4 = > 10 episodes) and the estimated volume of blood loss (0 = absent or negligible; 1 = ≤ 100 ml; 2 = > 100 ml). Information was obtained through interviews with patients and relatives, using a directed questionnaire. Mucorrhea and tenesmus were also assessed for clinical response to treatment. Patients were evaluated on the day of treatment initiation before the first infusion of dDAVP (day 0), on the next day (day 1), three days later (day 4), and in the final visit (day 14). Within 2–3 weeks or up to 4–5 weeks after beginning dDAVP treatment, patients underwent chemoradiotherapy or surgery, respectively.

### Tumor perfusion

Perfusion of rectal tumors was evaluated by dynamic contrast-enhanced magnetic resonance imaging (DCE-MRI) using a Philips Intera 1.5 T scanner and a Philips Achieva (Philips Medical Systems, The Netherlands). Images were acquired as a part of a standard clinical high-resolution rectal protocol, with a T2-weighted fast spin-echo images (section thickness, 3 mm), axial T1-weighted fast spin-echo images, axial free-breathing diffusion-weighted images (b values of 0, 800 and 1000 sec/mm^2^) and axial free-breathing images, T1-weighted fast field echo, 3D. Acquisition of DCE-MRI images of the entire tumor started 30 seconds before intravenous administration of 0.1 mM gadobutrol (0.1 ml of Gadovist, Bayer, Germany) per kg of body weight followed by a 20 mL saline flush at a rate of 2.0 mL/sec. Patients were evaluated before and on day 4 after treatment initiation with dDAVP, considering the area under the curve (AUC) as the main parameter for reduction of tumor perfusion.

### Biochemical analysis

Blood was drawn immediately before and 60 minutes after the first intravenous infusion of dDAVP. All laboratory assays were performed by investigators blinded to the clinical data, as described [22]. The VWF antigen (VWF:Ag) was measured by ELISA. The functional activity of VWF was analyzed through aggregometry by the von Willebrand ristocetin cofactor (VWF:RCo) assay, using fixed-washed platelets. The factor VIII levels (FVIII:C) were assayed applying the one-stage method. The standard pool was periodically calibrated against the WHO International Standard for FVIII and VWF in plasma (07/316). The activated partial thromboplastin time (APTT) and the euglobulin lysis time (ELT) were also determined as references for the effect of dDAVP on blood clotting and fibrinolysis, respectively.

### Statistical analysis

Statistical analysis was performed using Prism 6 statistical software (GraphPad, Inc. CA, USA). Results presented in this study were expressed as mean values ± SD or median value ± interquartile range. For ordinal qualitative variable (bleeding score) Friedman test followed by Dunn´s post-test was performed. For tumor perfusion and biochemical parameters (continuous quantitative variables) the difference between basal and post-treatment levels was analyzed by ratio paired t test, an adequate test for comparing differences between pairs when the effect depends on the initial measurement of the variable being analyzed. All statistically significant levels were defined as P < 0.05.

## Results

### Patients

A total of 44 patients was assessed for eligibility. Among these patients, 10 patients did not meet the inclusion criteria, while 2 refused to participate. The trial accrued 32 patients with bleeding rectal cancer from June 2013 to June 2017 at the Gastroenterology Hospital Bonorino Udaondo (n = 23) and the Alexander Fleming Institute (n = 9). Characteristics of the enrolled patients are summarized in Table 2. Median age at enrollment was 55.5 years. Most patients had moderate bleeding (90.6%), stage II or III disease (81.3%) and moderately differentiated adenocarcinoma histology (71.9%).

**Table 2:** Descriptive characteristics of patients enrolled in the study (n = 32)

Patient characteristic	No.
Sex	
Male	21 (65.6%)
Female	11 (34.4%)
Age, median (range)	55.5 years (19-75)
≥50 years	21 (65.6%)
<50 years	11 (34.4%)
Bleeding, median (interquartile range)	5 score points (4.2-6.0)
Moderate bleeding	29 (90.6%)
Severe bleeding	3 (9.4%)
Clinical TNM stage	
I	1 (3.1%)
II	6 (18.8%)
III	20 (62.5%)
IV	5 (15.6%)
Histopathology	
Adenocarcinoma	32 (100%)
Pathological differentiation	
Well differentiated	8 (25.0%)
Moderately differentiated	23 (71.9%)
Poorly differentiated	1 (3.1%)

### Safety and tolerability

Adverse events attributable to dDAVP were all reversible, and the majority were grade 1 or 2 (81.1%). The most frequent adverse events were hyponatremia, increased blood pressure, muscle cramps and facial flushing (Table 3). Five patients developed SAEs, including 1 of 6 patients from dose level group 3 (0.5 µg/kg/12 hours) showing hyponatremia (serum sodium levels < 130 mEq/L, grade 3) and increased blood pressure (systolic ≥ 160 or diastolic ≥ 100 mm Hg, grade 3), 2 of 6 patients from dose level group 4 (1 µg/kg/day) showing in both cases hyponatremia (grade 3), and 2 of 2 patients from dose level group 5 (1 µg/kg/12 hours) showing hyponatremia (grade 3) or hyponatremia (grade 3) and increased blood pressure (grade 3). No grade 4 events or treatment-related deaths were observed.

**Table 3:** Frequency of adverse events related to dDAVP administration

Adverse event	Number of events		
	Grade 1	Grade 2	Grade 3
Hyponatremia Increased blood pressure	5 4	0 3	5 2
Muscle cramps Facial flushing Hypokalemia Headache Increased transaminases Arthralgia Depressed level of consciousness Amnesia	5 4 2 2 1 1 1 1	1 0 0 0 0 0 0 0	0 0 0 0 0 0 0 0
Total	26 (70.3%)	4 (10.8%)	7 (18.9%)

DLT occurred in the 2 patients included in dose level group 5, and then in 2 of the 3 additional patients included in the next lower dose level group 4, although no DLT was observed in the 3 initial patients. Thus, the dose administered in dose level group 3 (0.5 µg/kg/12 hours) was determined as the MTD. Considering preliminary good results obtained for the hemostatic parameters with the first infusion of dDAVP at 0.5 µg/kg (see below), as well as the potential increased risk of hyponatremia with repeated doses at 12-hour intervals, we decided to select the dose of 0.5 µg/kg/day for further evaluation in the expansion cohort of 12 patients.

### Efficacy

**Fig. 1:**
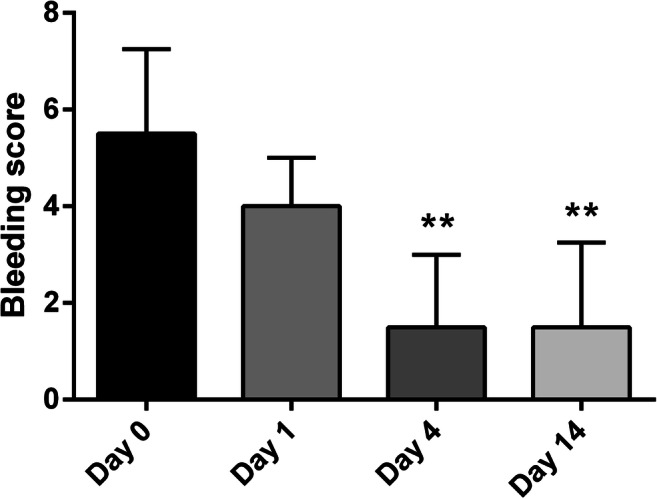
Bleeding score after dDAVP administration. Rectal bleeding was graded from 0 to 10 points using a score based on the Chutkan scale, as described in Methods section. Results obtained in patients from the expansion cohort (n = 12), receiving the selected daily dose of 0.5 µg/kg for two consecutive days, are shown. Patients were evaluated before the first infusion of dDAVP (day 0), one (day 1) and four (day 4) days later, and on the last follow-up (day 14). Data represent median ± interquartile range. **P < 0.01 versus day 0 (Friedman test followed by Dunn´s post-test).

Rectal bleeding was reduced in most patients, regardless of the dose of dDAVP administered (Table 4). About 90% (11/12) of patients from the expansion cohort receiving the selected dose of 0.5 µg/kg/day showed at least a PR. Moreover, almost 60% (7/12) of these patients developed CR with a total absence of bleeding symptoms at day 4 after initiation of dDAVP treatment and/or at the last follow-up at day 14. Accordingly, median bleeding score in these patients was significantly reduced at day 4, and remained low at the final visit at day 14 (Fig. 1). A similar trend was observed for accompanying symptoms, although severity of mucorrhea and tenesmus was mild in most patients at treatment initiation.

**Table 4:** Hemostatic response and effect on tumor perfusion after administration of dDAVP

Group	Hemostatic Response^a^		Reduction of tumor perfusion^b^
	PR + CR	CR	
Dose level 1 Dose level 2 Dose level 3 Dose level 4 Dose level 5	3/3 (100) 3/3 (100) 5/6 (83) 5/6 (83) NE^c^	2/3 (67) 3/3 (100) 4/6 (67) 5/6 (83) NE^c^	2/3 (67) 1/3 (33) 4/6 (67) 4/6 (67) NE^c^
Expansion cohort	11/12 (92)	7/12 (58)	8/12 (67)
Total	27/30 (90)	21/30 (70)	19/30 (63)

^a^Rectal bleeding was graded as absent, mild, moderate and severe. Responses were evaluated at day 4 after treatment initiation and/or at the last follow-up at day 14, as described in Methods section. ^b^Perfusion of rectal tumors was evaluated by DCE-RMI at day 4 after treatment initiation, considering AUC as the main parameter of reduction. ^c^Not evaluated, since the two patients included in this group rapidly developed SAE and treatment was discontinued. Data presented as n/total (%).

A preliminary evaluation of tumor perfusion by DCE-MRI showed that AUC was diminished at least 10% in 63% (19/30) of patients at day 4 after dDAVP administration considering all treatment groups (see also Table 4). Interestingly, in responder patients treated with the selected dose of 0.5 µg/kg/day a significant effect on tumor perfusion was observed, with a mean reduction of AUC of 23.9% (range 10.2 to 59%; P < 0.01 paired t test).

### Hemostatic parameters

As expected, VWF:Ag plasma levels exhibited a significant increase with respect to initial values after a first dDAVP infusion with all doses tested (Table 5). However, it is important to note that the lower dose of 0.25 µg/kg produced an increase of about 1.5-fold (50%), whereas with the doses of 0.5 and 1 µg/kg the increase was higher than 2-fold (120%). Similar results were found for VWF:RCo and FVIII:C levels, suggesting that a single dose of at least 0.5 µg/kg is required to produce a maximal hemostatic factor response in these patients. Likewise, modest, non-significant shortenings of APTT and ELT were observed using 0.25 µg/kg, but strong effects were found with higher doses of dDAVP (see also Table 5).

**Table 5:** Hemostatic parameters post-infusion of dDAVP

Dose^a^	VWF:Ag (%)^b^ Ref: 50–150		VWF:RCo (%)^b^ Ref: 50–150		FVIII:C (%)^b^ Ref: 50–150		APTT (seconds)^b^ Ref: 37–48		ELT (minutes)^b^ Ref: 90–240	
	Initial	Post dDAVP	Initial	Post dDAVP	Initial	Post dDAVP	Initial	Post dDAVP	Initial	Post dDAVP
0.25 µg/kg	167 ± 16.8	268 ± 28.1^*^	110 ± 11.4	156 ± 19.1^**^	115 ± 22.3	142 ± 30.7^**^	40 ± 2.7	37 ± 4.2	198 ± 31.0	134 ± 43.4
0.5 µg/kg 1.0 µg/kg	117 ± 14.2 143 ± 36.7	266 ± 19.0^***^ 309 ± 80.5^***^	83 ± 5.8 123 ± 36.4	170 ± 12.7^***^ 205 ± 33.0^*^	67 ± 8.4 106 ± 18.5	134 ± 17.1^***^ 276 ± 53.6^*^	61 ± 9.4 44 ± 3.0	43 ± 1.6^***^ 31 ± 3.1^**^	194 ± 16.2 208 ± 56.5	41 ± 5.3^***^ 40 ± 15.2^**^

^a^Dose level groups 1 + 2 (0.25 µg/kg) and dose level group 3 + expansion cohort group (0.5 µg/kg) are presented together, since in both cases patients received the same dose in the first infusion. Dose level group 4 (1.0 µg/kg) corresponds to the highest dose level analyzed, since the two patients included in dose level group 5 rapidly developed SAE and samples were not available for analysis of hemostatic parameters. ^b^VWF:Ag, VWF:RCo, FVIII:C, APTT and ELT were measured immediately before (initial) and 60 minutes after administration of the first intravenous treatment infusion (post dDAVP). The normal reference ranges (Ref) are presented for each parameter. In all cases, data represent mean ± SEM. *P < 0.05, **P < 0.01 and ***P < 0.001 vs the respective initial value (paired t test).

## Discussion

To our knowledge, this is the first clinical study of dDAVP as a hemostatic treatment in the management of rectal cancer. Although adverse events were reversible and mostly mild or moderate, DLT occurred in the 2 patients administered with the higher dose level 5 (1 µg/kg/12 hours), and then in 2 of the 3 additional patients receiving the next lower dose level 4 (1 µg/kg/day). DLT was also observed in 1 of 6 patients at dose level group 3 (0.5 µg/kg/12 hours). The most prominent treatment-related SAE was hyponatremia (serum sodium levels < 130 mEq/L), associated or not with increased blood pressure. In this regard, the risk of hyponatremia should be particularly regarded in elderly patients or after repeated doses of dDAVP with 12-hour intervals [23]. The MTD was determined to be 0.5 µg/kg/12 hours, and taking into consideration the good hemostatic response and safety of daily administrations, the dose of 0.5 µg/kg/day was selected for further evaluation in the expansion cohort.

In the present clinical trial, the hemostatic efficacy of dDAVP was prominent in most rectal cancer patients with bleeding, irrespective of the doses employed. Furthermore, about 60% of patients administered for two consecutive days with the selected dose of 0.5 µg/kg/day had complete absence of bleeding symptoms four days after treatment started. Such effect was sustained for at least ten additional days until the last follow-up appointment, clearly indicating that after a short, two-day dDAVP treatment the hemostatic response can remain for at least two weeks. This observation is important considering that the need of repeated administrations of dDAVP could exhaust storage sites of hemostatic factors, leading to tachyphylaxis [17].

Plasma levels of VWF and FVIII observed 60 minutes after the first dDAVP infusion clearly suggested that a single dose of at least 0.5 µg/kg is required to obtain maximal hemostatic factor responses in these patients, with increases of more than 100% in comparison to respective pretreatment values. These results are in agreement with early studies in healthy subjects [16], indicating that hemostatic factors increase rapidly in blood after release from endothelial storage sites. It is known that dDAVP acts via its strong AVPR2 agonist activity on vascular endothelium. This leads to exocytosis from Weibel Palade granules where VWF and FVIII are stored together with tissue-type plasminogen activator [7, 24]. In addition, a marked increase of platelet adhesiveness has been described as part of the hemostatic mechanisms exerted by dDAVP. Since the compound has no direct effects on platelets, it seems that dDAVP can induce the release of a platelet adhesion factor from endothelial cells, which is presumably VWF [25].

Regarding the studies by DCE-MRI, two-thirds of patients showed diminished tumor perfusion after treatment with dDAVP, with a reduction in the AUC of up to 59% in the expansion cohort receiving the selected dose of 0.5 µg/kg/day. Although very preliminary, these findings suggest a beneficial effect on tumor vascularization that could potentiate subsequent chemotherapy and radiotherapy. Experimental evidence has demonstrated a role of VWF in the modulation of angiogenesis, since its inhibition by short interfering RNA in endothelial cells caused increased angiogenesis, and VWF-deficient mice exhibited an enhanced vascularization response [26]. Besides, dDAVP was reported to reduce tumor-induced angiogenesis in vivo by favoring the formation of angiostatin through the proteolytic cleavage of plasminogen [12]. In preclinical colorectal cancer models, intravenous administration of clinically-relevant doses of dDAVP [11] or a second generation vasopressin analog [27] was capable of inhibiting tumor angiogenesis and impairing metastatic progression.

The reduction in tumor perfusion observed in several patients may suggest additional effects of dDAVP on tumor biology, and the potential favorable impact in subsequent specific oncologic treatment deserves further research. Repurposing of already-approved drugs, with a non-oncology primary purpose, might be an attractive approach to offer treatment options to patients with cancer. Drug repurposing allows faster development, reduced costs and lesser safety concerns as information on long-term pharmacovigilance for adverse effects is accessible [28]. At first glance, dDAVP looks promising from the results of this clinical study and previous in vitro and animal studies using colorectal cancer models [11].

There are some limitations of the present phase I/II trial. First, this was not a randomized, controlled study. Besides, the number of patients studied in each treatment group was small, thus limiting the power of the analysis of clinical outcomes. It should be take into account that bleeding can either be induced or aggravated by the diagnostic work-up of rectal cancer, and hemostasia is part of the natural response to bleeding once the diagnostic work-up has been completed. Therefore, hemostatic results, based on a symptomatic score, must be interpreted with caution as preliminary findings.

In conclusion, the vasopressin analog dDAVP appeared to be safe in rectal cancer patients with bleeding when administered in slow intravenous infusions at daily doses of 0.5 µg/kg during two consecutive days. Although this dosage has few side effects, the risk of hyponatremia should not be underestimated and fluids should be used with caution to avoid volume overload. The results of our study suggest that treatment is associated with hemostatic effects in most patients having moderate to severe rectal bleeding. We believe that the present phase I/II trial provides initial evidence supporting the use of dDAVP in the management of bleeding in patients with rectal cancer. Randomized phase III clinical trials to confirm the effectiveness of dDAVP as hemostatic option in rectal cancer, as well as in other gastrointestinal tumors, are warranted.

## Data Availability

Data generated or analyzed during this study are on file with Elea-Phoenix Laboratories and are not publicly available. Inquiries for data access may be sent to the following e-mail address: dileod@elea.com
